# Reliability and validity of the Children’s Sleep Habits Questionnaire—toddler in Chinese preschoolers

**DOI:** 10.3389/fpsyg.2026.1772030

**Published:** 2026-05-19

**Authors:** Yi Tang, Huijuan Peng, Songsong Huang, Fengjuan Li, Xiao Wang, Dan Huang, Jing Li, Xin Ding, Jing Hua

**Affiliations:** 1 Shanghai Key Laboratory of Maternal Fetal Medicine, Department of Women's and Children's Health Care, Shanghai First Maternity and Infant Hospital, School of Medicine, Tongji University, Shanghai, China; 2 School of Psychology, Shanghai University of Sport, Shanghai, China; 3 Shanghai Fourth People’s Hospital, School of Medicine, Tongji University, Shanghai, China; 4 JiaXing Maternity and Child Health Care Hospital, Jiaxing, China; 5 Children’s Hospital of Soochow University, Suzhou, China

**Keywords:** Children’s Sleep Habits Questionnaire, confirmatory factor analysis, exploratory factor analysis, reliability, toddlers, validity

## Abstract

**Objective:**

This study aims to evaluate the psychometric properties of the CSHQ-T and establish an empirically supported factor structure for assessing sleep habits among Chinese children aged 3 to 4 years based on a large population-based study.

**Methods:**

Caregivers of 37,556 children from multiple regions in China completed the online Chinese CSHQ-T. Reliability was assessed using internal consistency (Cronbach’s *α*), split-half reliability, and test–retest reliability (intraclass correlation coefficients, ICCs). Factor structure was examined through exploratory and confirmatory factor analyses (EFA, CFA). Concurrent validity was tested by correlating CSHQ-T scores with the Sleep Problems subscale of the Child Behavior Checklist for Ages 1½–5 (CBCL/1.5–5).

**Results:**

The total scale showed acceptable internal consistency (*α* = 0.769) and split-half reliability (Guttman coefficient = 0.857), with moderate test–retest reliability (ICCs = 0.66–0.78). EFA supported a four-factor structure—Sleep Distress, Sleep Duration, Sleep Initiation, and Sleep Transition—explaining 50.18% of the variance. CFA confirmed good model fit after excluding two items with factor loadings below 0.25 (GFI = 0.911, RMR = 0.026, RMSEA = 0.070). The CSHQ-T total score correlated moderately with the CBCL sleep subscale (*r* = 0.50, *p* < 0.01), supporting concurrent validity.

**Conclusion:**

The Chinese CSHQ-T demonstrates acceptable reliability and validity for assessing sleep habits in preschool children aged 3–4 years. The revised factor structure may be influenced by cultural practices, particularly the prevalence of co-sleeping in China. The instrument shows promise as a screening tool for pediatric sleep health in this population.

## Introduction

Sleep is a fundamental biological process, and has long been recognized as a key determinant of human health and performance (Perez-Pozuelo et al., 2020). Adequate sleep is particularly vital for children’s physical and mental well-being (Larsson et al., 2024), and insufficient or disrupted sleep in early childhood has been linked to behavioral and cognitive impairments (Gregory, 2012; Turnbull et al., 2013; Sadeh et al., 2014). Indeed, recent estimates indicate that roughly 25–45% of preschool and school-aged children experience significant sleep problems, which are in turn associated with poorer academic performance and social functioning (Mindell et al., 1999; Mindell and Meltzer, 2008; Kitamura, 2015). The preschool period represents a critical developmental period, a recent systematic review demonstrated that healthy sleep in this stage is associated with better behavioral and cognitive outcomes (Reynaud et al., 2018; Lionetti et al., 2021). However, despite its importance, research on sleep in preschool-aged children remains relatively limited (Bonuck et al., 2017). One major contributing factor to this gap is the lack of well-validated assessment tools specifically designed for this age group.

Various methods are available to assess pediatric sleep, ranging from objective measures such as polysomnography and actigraphy to subjective approaches including sleep diaries and parent-report questionnaires. Although objective methods are considered the gold standard, they require specialized equipment and controlled settings, making them costly and impractical for large-scale epidemiological or community-based studies (Ibáñez et al., 2018). By contrast, parent-report questionnaires are inexpensive, easy to administer to large samples, and capable of capture a child’s habitual sleep behavior in the home environment, albeit with the inherent limitations of self-report bias (Tikotzky and Sadeh, 2001). Consequently, standardized questionnaires remain a pragmatic and adopted option for sleep screening and epidemiological research in young children.

Among such instruments, the Children’s Sleep Habits Questionnaire (CSHQ) is one of the most widely utilized tools for assessing sleep behavior in children (Owens et al., 2000; Spruyt and Gozal, 2011). The original CSHQ was developed in the United States for school-aged children (4–10 years) (Bonuck et al., 2017) and was designed based on common pediatric sleep disorder symptoms. It comprises eight subscales addressing major domains such as bedtime resistance, sleep anxiety, night wakings, parasomnias, daytime sleepiness (Owens et al., 2000). To date, the CSHQ has been translated into at least 19 languages, and its psychometric properties have been evaluated across diverse international populations (Waumans et al., 2010; Silva et al., 2014; Fallahzadeh et al., 2015; Lucas-de La Cruz et al., 2016; Steur et al., 2017; Borrelli et al., 2021; Gios et al., 2022). A Chinese version of the CSHQ was previously validated by Li et al. (2007) and has been used in Chinese populations. It has been used in well over 300 studies worldwide (Bonuck et al., 2017). However, the vast majority of these validation studies have involved school-age samples, with relatively few investigations targeting preschool-aged children (Owens et al., 2000). Moreover, when the CSHQ has been applied to younger children, the original eight-factor structure has frequently failed to replicate (Owens et al., 2000). These findings suggest that the CSHQ in its original form may not adequately capture the unique sleep patterns of toddlers and preschoolers.

In response to these limitations, Sneddon et al. (2013) developed a modified version of the CSHQ for younger children, called the CSHQ-Toddler (CSHQ-T) (Sneddon et al., 2013). The CSHQ-T comprises 24 items and adopts a four-factor solution (Sleep Initiation, Sleep Distress, Transition to Waking, and Sleep Duration) that was empirically derived to better fit sleep behavior in 2–5-year-old children (Sneddon et al., 2013). Tan et al. (2018) conducted an initial evaluation of a Chinese translation of the CSHQ-T in a sample of 4–5-year-old children. Their findings indicated that certain subscales (e.g., Sleep Distress, Transition to Waking) demonstrated poor model fit, and that some factors failed to converge, suggesting problems with the factor structure in this sample (Tan et al., 2018). Notably, this study involved only 171 children, representing a relatively small sample that limits confidence in the stability and generalizability of the findings (Tan et al., 2018).

Given the limited validation of the CSHQ-T in Chinese preschoolers, the present study seeks to address this gap. Unlike previous Chinese studies that used the original 33-item CSHQ (Liu et al., 2014) or had small sample sizes (Tan et al., 2018; *N* = 171), this study is the first large-scale validation of the 24-item CSHQ-T in Chinese preschoolers. We did not perform a new translation; instead, we used the existing Chinese version of the CSHQ (Li et al., 2007) and mapped the 24-item CSHQ-T structure (Sneddon et al., 2013) onto it. The questionnaire was administered to a large, community-based sample of 3- to 4-year-old children in China. Specifically, we aimed to evaluate the questionnaire’s factor structure and examine its psychometric properties in this population. These analyses will help determine whether the Chinese CSHQ-T is a reliable and valid instrument for screening sleep habits and problems in this critical developmental age group within the Chinese context.

## Methods

### Participants

**Figure 1:**
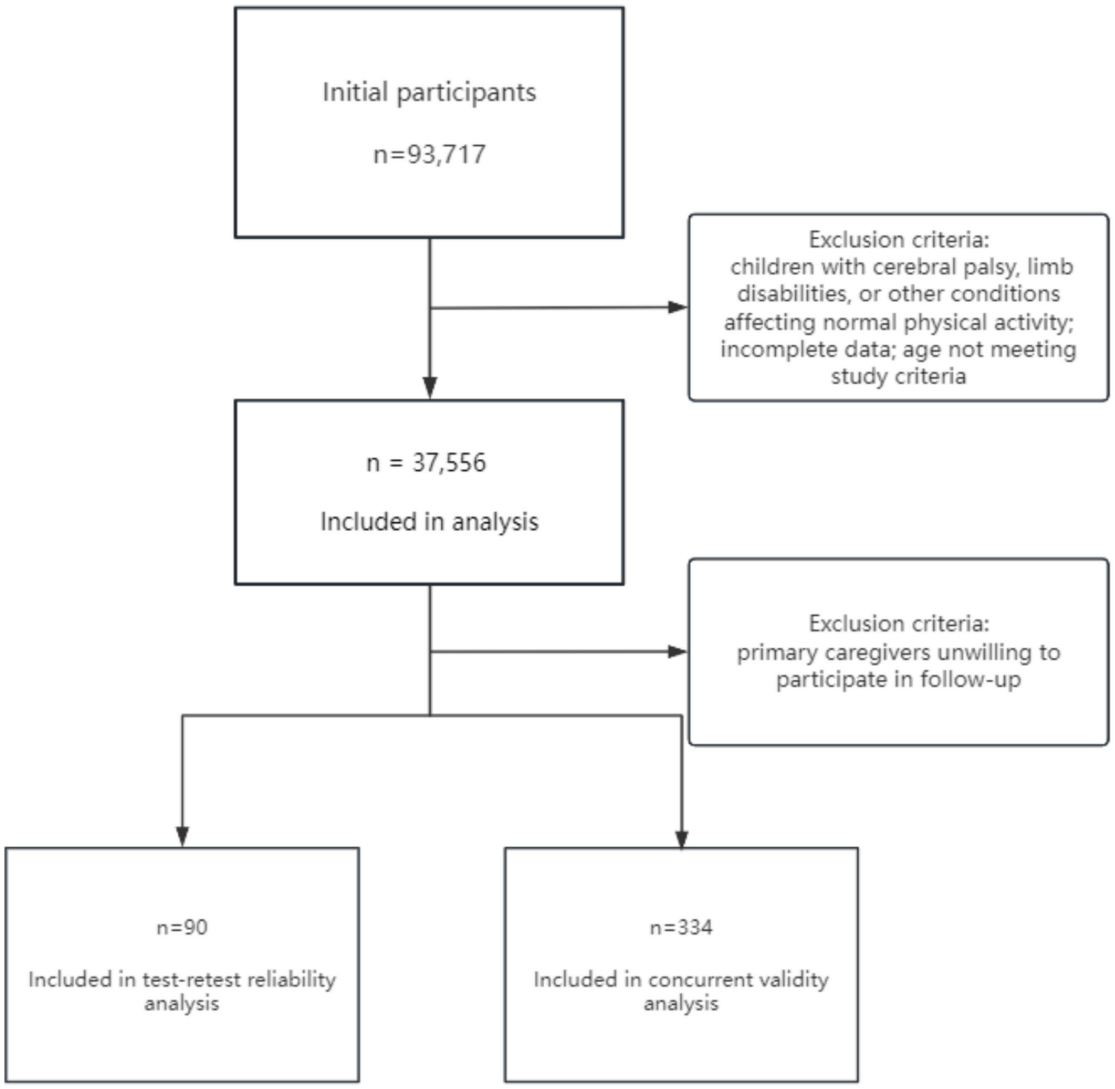
Participant flow diagram.

Caregivers of children aged 3–4 years were recruited nationwide through the Shanghai First Maternity and Infant Hospital’s online child health platform. Eligible participants were 3–4 years old with typical cognitive development. To ensure a healthy sample, exclusion criteria included congenital or acquired neurological or intellectual disability (e.g., cerebral palsy), severe physical impairments. Caregivers were excluded if they were unable or unwilling to complete the questionnaire or if the submitted data were incomplete (see Figure 1). Data were collected between April 2018 and December 2019 from multiple regions across China (e.g., Songjiang District, Shanghai; Yangzhou, Jiangsu; Shaoxing and Wenzhou, Zhejiang; Weinan and Xianyang, Shaanxi; Haikou, Hainan; Xiamen, Fujian). A subsample of participants in Shanghai and Jiaxing (Zhejiang Province) was invited to complete the survey again to assess test–retest reliability and concurrent validity. The study protocol was approved by the Ethics Committee of Tongji University, and informed consent was obtained from all parents or legal guardians prior to participation.

### Measures

#### Children’s Sleep Habits Questionnaire – Toddler (CSHQ-T), Chinese version

The original Children’s Sleep Habits Questionnaire (CSHQ) was developed in English by Owens et al. (2000). A Chinese version of the CSHQ was subsequently developed and validated by Li et al. (2007) through forward-backward translation. Later, Sneddon et al. (2013) developed the CSHQ-Toddler (CSHQ-T) by selecting a subset of 24 items from the original English CSHQ based on factor analysis, without altering the wording of the retained items. In the present study, we adopted the 24-item structure of the CSHQ-T [i.e., the specific items identified by Sneddon et al. (2013)] and mapped these items onto the existing Chinese translation of the CSHQ developed by Li et al. (2007). No new forward-backward translation was performed, because the Chinese wording of all 24 CSHQ-T items was already available in the Li et al. (2007) Chinese version. Thus, the questionnaire administered in this study consists of those 24 items from Li et al. (2007), organized into four subscales as defined by Sneddon et al. (2013): Sleep Distress, Sleep Duration, Sleep Transition, and Sleep Initiation. Caregivers rated each behavior on a 3-point frequency scale (1 = “occasionally,” 2 = “sometimes,” 3 = “often”). Item scores were summed to yield a total sleep problem score (range 24–72), with higher scores reflecting more frequent or severe sleep problems.

#### Child behavior checklist for ages 1½–5 (CBCL/1.5–5)

As an external validation measure, the Sleep Problems subscale of the Chinese CBCL/1.5–5 was employed. The CBCL/1.5–5 is a widely used behavioral assessment for preschoolers, and its Sleep Problems subscale contains six items relating to common sleep issues. These items ask about behaviors such as nightmares, difficulty falling asleep, and frequent night awakenings over the past 6 months. Each item is rated on a 3-point scale (0 = “not true,” 1 = “somewhat or sometimes true,” 2 = “very true or often true”), consistent with the standard CBCL scoring format. Subscale score were calculated by summing the six item responses. The Chinese version of the CBCL/1.5–5 has been empirically validated and exhibits good reliability in Chinese preschool samples. In the present study, CBCL Sleep Problems scores served as a criterion measure for evaluating concurrent validity.

#### Cultural adaptation

The Chinese CSHQ-T used in this study underwent a formal cultural appropriateness review. A panel of three pediatric sleep specialists and five parents assessed the items for clarity and cultural relevance. Item retention decisions were guided by factor loadings in EFA (loading < 0.25 as removal criterion), not by subjective cultural judgments. Two solitary-sleep items were removed due to low loadings, consistent with empirical criteria.

### Procedure

After obtaining informed consent, primary caregivers completed the questionnaires online via the hospital’s child health platform. The survey included the Chinese CSHQ-T and the CBCL/1.5–5 Sleep Problems subscale, and was administered once between April 2018 and December 2019. To assess test–retest reliability, the researchers recontacted a subset of caregivers from Shanghai and Jiaxing, randomly selecting participants who were willing to take part in the follow-up, and asked them to complete the CSHQ-T questionnaire again approximately 2 weeks later. This interval was selected to be sufficiently long to minimize recall while remaining short enough to reduce the likelihood of genuine changes in children’s sleep patterns. All data were collected securely either electronically via the study platform or using paper-based questionnaires.

### Data analysis

The psychometric properties of the CSHQ-T were evaluated using a multi-step analytic approach. First, an exploratory factor analysis (EFA) was conducted to examine the underlying structure of the questionnaire. Items with factor loadings below 0.25 in the EFA were considered for removal, based on conventional criteria for weak loadings. Item-total correlations were examined for descriptive purposes only and were not used as a deletion criterion. The number of factors was determined based on both statistical criteria (eigenvalues > 1, scree plot) and the conceptual framework of toddler sleep symptoms. Following the EFA, a confirmatory factor analysis (CFA) was performed to test the established factor model. Model fit was assessed using the Goodness-of-Fit Index (GFI), the Root Mean Square Residual (RMR), and the Root Mean Square Error of Approximation (RMSEA). Acceptable model fit was defined as GFI > 0.90, RMR < 0.08, and RMSEA < 0.08, following established guidelines (Browne and Cudeck, 1992; Hu and Bentler, 1999).

In addition, the internal consistency of the CSHQ-T was examined using Cronbach’s *α* coefficients for both the total scale and individual subscales; split-half reliability was also assessed using an odd-even item method. Test–retest reliability was evaluated through the computation of intraclass correlation coefficients (ICCs) between scores derived from two assessment waves administered with an approximately two-week interval (consistent with the procedure described in the Procedure section). Concurrent validity was assessed by examining Pearson correlation coefficients between CSHQ-T scores and scores on the Sleep Problems subscale of the CBCL/1.5–5.

Given the large sample size (*n* = 37,556), even trivial effects can achieve statistical significance at *p* < 0.05. Therefore, we focused on effect sizes and model fit indices as primary evidence for validity, rather than relying solely on *p*-values. For the concurrent validity analyses (Pearson correlations), we set a more conservative alpha level of *p* < 0.01 to reduce the risk of Type I error. Multiple comparisons were not adjusted further because the main analyses (EFA, CFA, reliability) are not hypothesis tests in the conventional sense; however, we acknowledge this as a limitation in the Discussion.

All statistical analyses were performed using SPSS version 24.0 and AMOS version 28.0.

## Results

### Sample characteristics

A total of 37,556 preschool children (aged 3–4 years) were included in the analysis after excluding cases with age mismatches or logical inconsistencies. The mean age of the sample was 3.96 years (SD = 0.55), and the mean age in months was 47.53 months (SD = 6.58). Boys accounted for 47.8% of the sample. The mean total CSHQ-T score was 32.99 (SD = 5.80). Additional sociodemographic details—including parental education levels, family structure, and relevant clinical information—are presented in Table 1, confirming the robustness and geographical diversity of the sample.

**Table 1:** Children’s demographic and health characteristics of subjects (*n* = 37,556).

Variables	*n*	%
Sex		
Male	17,965	47.8
Female	19,591	52.2
Age		
3 years old	19,405	51.7
4 years old	18,151	48.3
Preterm		
Yes	5,533	14.7
No	32,023	85.3
Single-child family		
Yes	17,170	45.7
No	20,386	54.3
Higher education of mother		
Yes	16,919	45.1
No	20,637	54.9
Higher education of father		
Yes	16,315	43.4
No	21,241	56.6
Taking psychotropic medications		
Yes	310	0.8
No	37,246	99.2
Family structure		
Nuclear family	22,644	60.3
Immediate family	12,140	32.3
United family	1863	5
Single parent family	909	2.4

“Higher education” refers to completion of college or university education (associate’s degree or bachelor’s degree or higher). Family structure definitions: Nuclear family = child lives with both parents only; Immediate family = child lives with parents and grandparents; United family = child lives with grandparents, parents, parents’ siblings, and the child; Single parent family = child lives with only one parent. “Psychotropic medications” include psychostimulants, anticonvulsants, antihistamines with sedative effects, and other medications prescribed for the child that may affect sleep.

### Reliability

The internal consistency of the 22-item Chinese version of the CSHQ-T was acceptable. The overall Cronbach’s *α* for the total scale was 0.769, and item-deletion analysis revealed that no single item disproportionately influenced the scale’s internal consistency (see Table 2). Subscale reliabilities varied, with the Sleep Distress subscale demonstrating good consistency (*α* = 0.822), while the Sleep Duration (*α* = 0.699), Sleep Transition (*α* = 0.781), and Sleep Initiation (*α* = 0.652) subscales showed acceptable to moderate levels of reliability. In addition, the Guttman split-half coefficient was 0.857, further supporting the overall reliability of the instrument. Test–retest reliability, as measured by intraclass correlation coefficients (ICCs = 0.66–0.78), indicated moderate temporal stability (see Table 3).

**Table 2:** Internal consistency of CSHQ-T (*n* = 37,556).

Item no.	Item content	Mean	SD	Item total correlations	Cronbach *α* coefficient after deletion of items
1	Goes to bed at same time	1.45	0.645	0.157	0.752
2	Asleep in 20 min	1.68	0.707	0.161	0.752
5	Needs parent in room	2.42	0.742	0.173	0.752
6	Struggles at bed time	1.33	0.584	0.437	0.735
7	Afraid in dark	1.57	0.773	0.348	0.739
8	Afraid alone	1.97	0.834	0.332	0.741
9	Sleeps too little	1.54	0.678	0.394	0.736
10	Sleeps right amount	1.76	0.775	0.061	0.761
11	Sleeps same amount	1.58	0.730	0.084	0.758
12	Talks during sleep	1.28	0.513	0.419	0.737
13	Rest less during sleep	1.51	0.647	0.392	0.737
14	Moves to other bed	1.20	0.485	0.396	0.739
15	Trouble away from home	1.29	0.570	0.349	0.740
16	Awakens screaming	1.19	0.452	0.501	0.735
17	Alarmed by scary dream	1.16	0.418	0.510	0.735
18	Awakens once in night	1.27	0.535	0.396	0.738
19	Awakens more in night	1.18	0.455	0.451	0.737
20	Wakes by him/herself	1.79	0.786	0.038	0.763
21	Wakes in negative mood	1.37	0.563	0.479	0.733
22	Others wake child	1.70	0.707	0.377	0.737
23	Difficulty getting up	1.44	0.628	0.509	0.729
24	Long time to become alert	1.31	0.540	0.532	0.730

Cronbach’s *α* coefficients are rounded to three decimal places. Due to rounding, some values appear identical (e.g., 0.752), but the actual calculated values differ slightly (e.g., 0.7523 vs. 0.7518).

**Table 3:** Test–retest reliability of CSHQ-T (*n* = 90).

Factor name	ICC	95% CI
Sleep distress	0.705	0.584, 0.796
Sleep duration	0.736	0.624, 0.818
Sleep initiation	0.661	0.527, 0.763
Sleep transition	0.777	0.679, 0.847
Total score	0.724	0.608, 0.809

### Exploratory factor analysis (EFA)

Preliminary analyses confirmed the suitability of the data for factor analysis (Kaiser–Meyer–Olkin = 0.869; Bartlett’s test of sphericity: *χ*^2^ = 239,258.40, *p* < 0.001). A four-factor solution was extracted, accounting for 50.18% of the total variance. These factors aligned well with the hypothesized sleep domains–Sleep Distress, Sleep Duration, Sleep Transition, and Sleep Initiation. The factor loadings for each items are presented in Table 4, showing that items predominantly loaded onto their expected factor. The factor loadings in Table 4 are empirically derived. Some assignments may appear counterintuitive (e.g., “asleep in 20 minutes” loading on Sleep Duration rather than Sleep Initiation). This likely reflects that Chinese caregivers in our sample perceived quick sleep onset as an indicator of overall sleep adequacy rather than a separate initiation problem. Similarly, “sleeps too little” loaded on Sleep Initiation, possibly because perceived sleep insufficiency was linked to bedtime resistance. These interpretations are preliminary and require replication.

**Table 4:** Factor loadings of the CSHQ-T items based on the exploratory factor analysis (*n* = 37,556).

Item no.	Item content	Sleep distress	Sleep duration	Sleep initiation	Sleep transition
Sleep distress (*α* = 0.822)					
12	Talks during sleep	0.623			
13	Restless during sleep	0.518			
14	Moves to other bed	0.583			
15	Trouble away from home	0.504			
16	Awakens screaming	0.743			
17	Alarmed by scary dream	0.777			
18	Awakens once in night	0.715			
19	Awakens more in night	0.752			
Sleep duration (*α* = 0.699)					
1	Goes to bed at same time		0.729		
2	Asleep in 20 min		0.604		
10	Sleeps right amount		0.718		
11	Sleeps same amount		0.771		
20	Wakes by him/herself		0.487		
Sleep initiation (*α* = 0.652)					
5	Needs parent in room			0.623	
6	Struggles at bedtime			0.431	
7	Afraid in dark			0.686	
8	Afraid alone			0.796	
9	Sleeps too little			0.406	
Sleep transition (*α* = 0.781)					
21	Wakes in negative mood				0.542
22	Others wake child				0.798
23	Difficulty getting up				0.820
24	Long time to become alert				0.695

Extraction method-Principal Component Analysis; Factor rotation method-Maximum Variance method.

### Confirmatory factor analysis (CFA)

**Figure 2:**
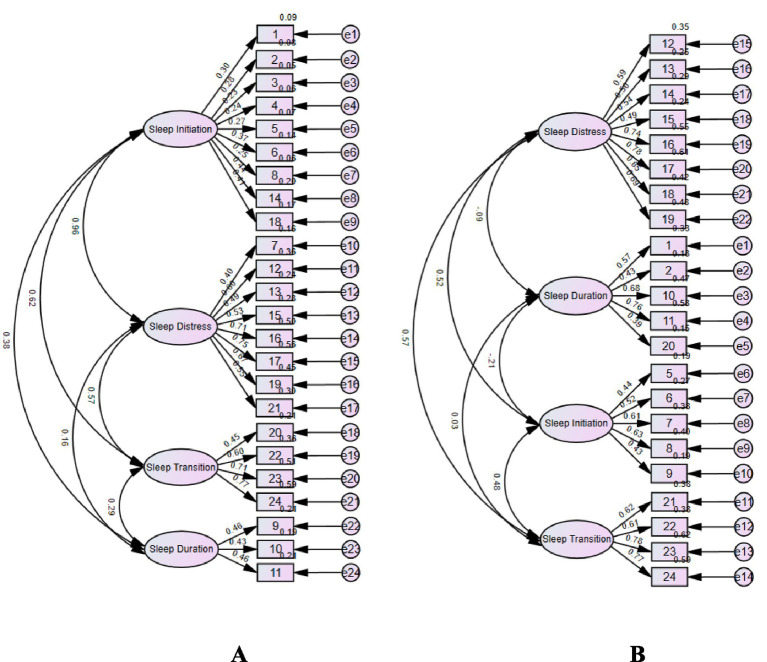
Initial **(A)** and modified **(B)** structural model of CSHQ-T. Numbers inside rectangles refer to item numbers as listed in Table 4. Modified model **(B)** excludes two items (item 3 and 4) due to factor loadings < 0.25. For factor abbreviations and fit indices, see Results section.

An initial CFA evaluating the theoretically derived four-factor model revealed suboptimal fit indices (GFI = 0.747; RMR = 0.063; RMSEA = 0.107), indicating that the original model did not adequately capture the structure of sleep in this sample (see Figure 2). Guided by the EFA findings, two items (“falls asleep alone in own bed” and “falls asleep in another’s bed”) with factor loadings below 0.25 were removed. The revised model exhibited substantially improved fit (GFI = 0.911; RMR = 0.026; RMSEA = 0.070), meeting the pre-established criteria for model adequacy (GFI > 0.90, RMR < 0.08, RMSEA < 0.08), as depicted in Figure 2.

### Concurrent validity

To assess convergent validity, Pearson correlation coefficients were calculated between the CSHQ-T and the Sleep Problems subscale of the CBCL/1.5–5 (*n* = 334). The total score of the CSHQ-T was moderately strongly correlated with the CBCL sleep measure (*r* = 0.50, *p* < 0.01), which supports the convergent validity of the instrument. The Pearson correlation coefficients for the “Sleep Duration,” “Sleep Initiation” and “Sleep Distress” calculated by the CSHQ-T and CBCL sleep problems subscale were 0.395 (*p* < 0.01), 0.329 (*p* < 0.01) and 0.328 (*p* < 0.01), respectively. However, the Pearson’s coefficients for “Sleep Transition” was 0.182 (*p* < 0.01). In contrast, correlations between the individual CSHQ-T subscales and the CBCL subscale ranged from 0.182 to 0.395. Although these associations were statistically significant, their modest magnitudes suggest that subscale scores should be interpreted cautiously when used independently. Overall, these findings indicate that the Chinese CSHQ-T is a reliable and valid tool for screening sleep problems in preschool children.

To assess concurrent validity, Pearson correlation coefficients were calculated between the CSHQ-T total and subscale scores and the Sleep Problems subscale of the CBCL/1.5–5. The CSHQ-T total score showed a moderate correlation with the CBCL sleep subscale (*r* = 0.50, *p* < 0.01), which is comparable to those reported in previous validation studies of the CSHQ in other populations. For example, Sneddon et al. (2013) reported correlations of 0.09–0.51 between CSHQ-T subscales and CBCL sleep problems. Similarly, Lionetti et al. (2021) found that CSHQ total scores distinguished children with borderline/clinical sleep problems from those without. The correlations for individual CSHQ-T subscales with the CBCL sleep subscale were: Sleep Duration (*r* = 0.395, *p* < 0.01), Sleep Initiation (*r* = 0.329, *p* < 0.01), Sleep Distress (*r* = 0.328, *p* < 0.01), and Sleep Transition (*r* = 0.182, *p* < 0.01). These moderate to modest correlations are expected because the CBCL sleep subscale measures a global perception of sleep problems using only six items, whereas the CSHQ-T provides a multi-dimensional assessment across 24 items covering specific behaviors. The weaker correlation for the Sleep Transition subscale (which captures morning awakening behaviors) is consistent with Sneddon et al. (2013), who similarly found that this domain was less strongly associated with the CBCL sleep measure. Taken together, these results support the concurrent validity of the CSHQ-T total score, but subscale scores, particularly Sleep Transition, should be interpreted with caution.

## Discussion

Based on a large population-based sample, this study provides the first evidence that the Chinese version of the Children’s Sleep Habits Questionnaire–Toddler (CSHQ-T) demonstrates overall acceptable reliability and validity in 3–4-year-old children. Internal consistency (Cronbach’s *α*) and split-half reliability for the total score were within acceptable ranges, indicating adequate internal coherence. Test–retest reliability was satisfactory, suggesting stable measurement of sleep problems over time. Exploratory and confirmatory factor analyses supported a four-factor structure, which, although interpretable, diverged from the original four-domain model. Concurrent validity was supported by modest yet significant correlations with a validated behavioral sleep-problem measure (CBCL/1.5–5 sleep scale). Collectively, these findings suggest that the Chinese CSHQ-T has acceptable reliability and moderate concurrent validity for screening sleep disturbances in preschool children, though its subscale configuration may not fully correspond to that of the original version. Subscale scores, particularly for Sleep Transition, should be interpreted with caution.

### Comparison with prior validation studies

In our large sample, the internal consistency of the CSHQ-T total scale and subscales ranged from acceptable to good. Although slightly lower than that reported in the original validation study (Sneddon et al., 2013), which included only 105 participants, our findings are based on 37,556 cases, providing substantially greater representativeness. Test–retest reliability of the total scale and subscales ranged from moderate to acceptable, and split-half reliability was also satisfactory. Notably, the original version did not assess these indices, further underscoring the robustness of our reliability results. The observed stability of the CSHQ-T is consistent with earlier pilot studies that reported reasonable test–retest agreement in both toddlers (Goodlin-Jones et al., 2008) and preschoolers (Sneddon et al., 2013).

Regarding factor structure, our four-factor solution parallels findings from several prior validations. Sneddon et al. (2013) similarly reported that the original 33-item, eight-domain structure did not fit well in a 2–5-year-old sample, instead identifying a four-factor model. Dias et al. (2018) also found four factors (e.g., bedtime resistance, sleep anxiety, positive sleep habits, daytime sleepiness) in a Portuguese infant version of the CSHQ. Lionetti et al. (2021) reported seven factors for Italian children aged 3–6 years, noting substantial divergence from the original structure. In contrast, Liu et al. (2014), using a large Chinese preschool cohort, proposed a modified eight-factor model that retained the original domains but reassigned or removed certain items to improve model fit. Together with our findings, these studies suggest a consistent pattern: when applied to younger children or across cultural contexts, the CSHQ often requires empirical modification. In our study, the final four factors captured the core dimensions of sleep problems among Chinese preschoolers, though they did not map precisely onto the original eight domains.

Concurrent validity was also supported. The CSHQ-T total score and subscales correlated significantly with the CBCL/1.5–5 Sleep Problems subscale. However, the Sleep Transition subscale consistently showed weaker correlations, a finding also noted in the original CSHQ-T study with the CBCL/1.5–5 (Sneddon et al., 2013). This suggests that, although its convergent validity is limited, the Sleep Transition subscale may provide complementary insights into sleep–wake cycle regulation not captured by other measures.

The CBCL/1.5–5 Sleep Problems subscale is a brief screening tool consisting of six items (e.g., “difficulty falling asleep,” “nightmares,” “wakes frequently”). It is useful for identifying children who may have general sleep difficulties, but it does not differentiate among specific types of sleep problems. In contrast, the CSHQ-T provides a domain-specific profile across four empirically derived dimensions: Sleep Distress (e.g., night wakings, parasomnias), Sleep Duration (regularity and adequacy of sleep), Sleep Initiation (bedtime resistance and fears), and Sleep Transition (morning awakening behaviors). This granularity is valuable for both research and clinical practice. For example, a child with high Sleep Initiation scores may benefit from behavioral interventions targeting bedtime resistance, whereas a child with high Sleep Distress scores may require assessment for night terrors or sleep apnea. The moderate correlation (*r* = 0.50) between the two instruments indicates that they measure related but distinct constructs; the CSHQ-T complements rather than replaces the CBCL sleep subscale. Therefore, the CSHQ-T is particularly suited for studies or clinical evaluations that require a detailed characterization of sleep behaviors in preschoolers.

Several validation studies have reported four-factor solutions for the CSHQ or CSHQ-T in young children, but the composition of these factors varies. Sneddon et al. (2013) identified four factors: Sleep Initiation, Sleep Distress, Sleep Duration, and Transition to Waking. Our factors are similarly labeled but differ in item composition. For instance, “asleep in 20 minutes” loaded onto Sleep Initiation in Sneddon’s study, whereas in our sample it loaded onto Sleep Duration. Dias et al. (2018) extracted four factors (Bedtime Resistance, Sleep Anxiety, Positive Sleep Habits, Daytime Sleepiness) from the CSHQ-Infant, which only partially overlap with ours. Lionetti et al. (2021) reported seven factors in Italian preschoolers, with “Bedtime Resistance” and “Night Wakings” as separate domains, while these merged into a broader Sleep Distress factor in our sample. These discrepancies likely reflect developmental differences and cultural variations in sleep practices (e.g., co-sleeping prevalence in China). Therefore, factor numbers and labels should not be directly compared across studies without examining item-level loadings; instead, the empirical structure should be interpreted within each specific sample and cultural context.

### Cultural considerations

Cultural context may help explain the differences observed in factor structure and item performance. Significant cultural variations exist between China and other countries, and these differences strongly influence children’s sleep behaviors (Jiang et al., 2016). For example, co-sleeping (bed-sharing) is common in Chinese families. Jiang et al. (2016) reported that bed-sharing is highly prevalent among Chinese children and adolescents (16.8% in a sample aged 9–13 years), with parental positive attitude being the most determining factor. For preschool-aged children, the prevalence is even higher. In our sample, the two items related to solitary sleeping (“falls asleep alone in own bed” and “falls asleep in another’s bed”) had very low factor loadings (<0.25) and were therefore removed on empirical grounds. This finding is consistent with the cultural context in which solitary sleeping is less common and thus may not meaningfully differentiate children with versus without sleep problems. However, we acknowledge that co-sleeping being normative does not automatically mean it is never a problem; rather, its clinical significance depends on the presence of other sleep difficulties (e.g., frequent night wakings, bedtime resistance). Therefore, we caution against interpreting co-sleeping alone as a sleep problem indicator in Chinese children. Instead, clinicians should consider co-sleeping within the broader pattern of sleep behaviors. Future cross-cultural studies should formally test measurement invariance of the CSHQ-T across different cultural groups.

### Item content considerations

The CSHQ-T includes several pairs of items that may appear contradictory at first glance, such as “sleeps too little” (item 9) and “sleeps right amount” (item 10), or “awakens once in night” (item 18) and “awakens more in night” (item 19). These items are not intended to be mutually exclusive; rather, they assess different facets of sleep perception. For example, a caregiver might rate a child as both “sleeping too little” (indicating perceived insufficiency) and “sleeping the right amount” (indicating satisfaction with sleep duration) depending on the reference point. Similarly, a child who wakes twice per night would receive a rating of “sometimes” or “often” on both “awakens once” and “awakens more” items, capturing frequency patterns. These item pairs were retained from the original CSHQ-T (Sneddon et al., 2013) because they provide clinically useful information. However, future refinements could consider merging or rephrasing these items to reduce potential confusion for respondents.

### Limitations

Several limitations should be acknowledged. First, sleep problems were assessed exclusively through parent report, which may be subject to reporting bias; no objective measures (e.g., actigraphy or direct observation) were employed to verify parental perceptions against actual sleep parameters. Second, although geographically diverse across 11 provinces, the sample comprised generally healthy community preschoolers, thereby limiting generalizability to clinical populations such as children with developmental disorders or chronic illnesses. Third, the cross-sectional design precluded examination of the long-term stability of the factor structure and scores across developmental stages. Fourth, while linguistic accuracy of the translation was ensured, measurement invariance was not formally tested. Fifth, the exploratory factor analysis (EFA) and confirmatory factor analysis (CFA) were conducted on the same large sample (*n* = 37,556). Although large sample sizes reduce sampling error, using the same dataset for both model generation (EFA) and model testing (CFA) may lead to overfitting and inflated fit statistics for the derived model. Ideally, the sample would be split into a calibration subsample for EFA and a validation subsample for CFA, or an independent replication sample would be used. Therefore, our CFA results should be interpreted as preliminary, and future studies are needed to validate the proposed four-factor structure in an independent Chinese preschool sample. Finally, certain items (e.g., enuresis) were infrequent in this age group and thus effectively excluded from analysis, highlighting the need for establishing normative values for Chinese preschoolers. These issues warrant further investigation in future research.

### Implications for practice

Despite limitations, the present findings offer preliminary guidance for using the CSHQ-T in Chinese preschoolers. The total score demonstrates acceptable internal consistency and moderate test–retest reliability, suggesting that it may be suitable for group-level comparisons and initial screening in community or epidemiological research. However, several caveats apply. First, the moderate concurrent validity (*r* = 0.50) and the modest reliability of some subscales (e.g., Sleep Initiation, *α* = 0.652) indicate that the CSHQ-T is not yet ready for individual clinical diagnosis without further validation. Second, clinical cutoff scores have not been established for Chinese children; therefore, the instrument should be used to flag potential sleep difficulties rather than to confirm a diagnosis. Third, the Sleep Transition subscale showed a weak correlation with the CBCL sleep measure (*r* = 0.182) and should be interpreted with particular caution, or omitted if the focus is on concurrent validity against the CBCL. Pediatricians and early childhood practitioners may use the CSHQ-T as a brief, low-cost screening aid to identify children who warrant more comprehensive assessment (e.g., clinical interview, actigraphy, or sleep diary). High total scores could trigger referral for sleep hygiene guidance or further evaluation, but decisions should not be based solely on questionnaire scores. Future research should establish population-based normative values and test the predictive validity of the CSHQ-T against objective sleep measures and clinical diagnoses.

### Future research directions

Future studies should extend this initial validation in several important ways. Longitudinal research is needed to assess the stability of the CSHQ-T factor structure over time and to determine whether early sleep problem scores predict later outcomes, such as emotional or cognitive development. Testing the scale in clinical populations (e.g., children with ADHD, autism, or chronic illness) would help establish its discriminative validity and utility in high-risk groups. Cross-cultural replications, particularly in other East Asian societies, could clarify which findings are culture-specific versus generalizable. In addition, future work should establish normative values or cutoff scores for Chinese preschoolers, with thresholds calibrated to population distributions. Finally, integrating parent-report with objective measures such as actigraphy or polysomnography would enhance criterion validity and support refinement of scale items.

## Conclusion

The Chinese CSHQ-T demonstrates acceptable psychometric properties for assessing sleep in preschool children aged 3–4 years. The total score shows acceptable internal consistency and test–retest reliability, with moderate concurrent validity relative to an established sleep measure. The factor structure of the Chinese CSHQ-T diverges from the original four-factor model reported by Sneddon et al. (2013). This divergence may be influenced by a combination of factors, including the developmental stage of the sample (3–4 years vs. the original 2–5 years), cultural differences in sleep practices (e.g., co-sleeping prevalence in China), and potential translation effects. However, these factors are inherently confounded in cross-cultural adaptation studies, and we cannot definitively attribute the observed divergence to any single cause. Subscale scores, particularly for Sleep Transition, require cautious interpretation. Overall, the Chinese CSHQ-T represents a promising screening tool for pediatric sleep health in Chinese preschoolers, pending further validation in clinical populations and against objective sleep measures.

## Data Availability

The original contributions presented in the study are included in the article/supplementary material, further inquiries can be directed to the corresponding authors.
